# P-933. Combination Antibiotic Therapy in Infective Endocarditis: A systematic Review and Meta Analysis

**DOI:** 10.1093/ofid/ofae631.1124

**Published:** 2025-01-29

**Authors:** Parisa Farahani, Felicia Ruffin, Mohammad Taherahmadi, Maren E Webster, Rachel E Korn, Sarah Cantrell, Lana Wahid, Vance G Fowler, Joshua T Thaden

**Affiliations:** Department of Medicine, Duke University, Roanoke, Virginia; Duke University Medical Center, Durham, NC; Tehran University of Medical Science, Arak, Markazi, Iran; Duke University Medical Center, Durham, NC; Duke University Medical Center, Durham, NC; Duke University, Durham, North Carolina; Carilion Clinic; Virginia Tech Carilion School of Medicine, Roanoke, Virginia; Duke University Medical Center, Durham, NC; Duke University School of Medicine, Durham, NC

## Abstract

**Background:**

Infective endocarditis (IE) is serious disease with 20% in-hospital mortality and significant morbidity. The role of combination therapy in managing patients with IE is controversial. We aimed to determine the effectiveness of combination antibiotic therapy compared to monotherapy in IE.

Preferred Reporting Items for Systematic Reviews and Meta-Analyses (PRISMA) flow diagram; summarizes the study selection process

**Figure:**
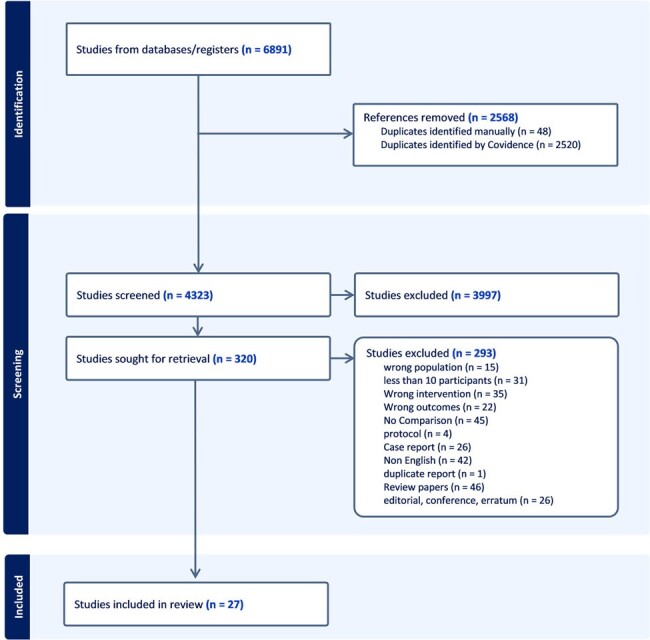

**Methods:**

MEDLINE, Embase, Cochrane and Web of Science were searched from inception to July 28, 2023. Papers reporting mortality of combination therapy versus monotherapy in adult patients with IE were included. Non-English papers and studies with < 10 patients in combination therapy group were excluded. Two reviewers independently assessed the studies. Summary of odds ratios (ORs) and 95% confidence intervals (CIs) were evaluated using a random-effects model. Sub-group analyses based on timing of mortality, bacterial species/group, geographic location, and study design were conducted.

Forest Plot of all-cause Mortality at any time point of patients with infective endocarditis compared between combination therapy and monotherapy

**Figure:**
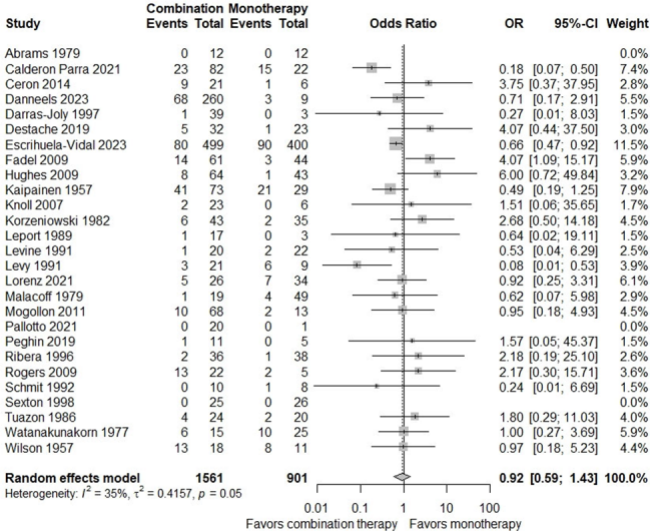

**Results:**

Out of 4323 studies identified, 27 studies (2462 patients) were included in the meta-analysis. There was no significant difference in the risk of all-cause mortality between the two groups (OR, 0.92; 95% CI, 0.59-1.43). Subgroup analysis focusing on one-year mortality demonstrated a decrease in mortality risk with combination therapy (OR, 0.54; 95% CI, 0.33-0.87). Additionally, combination therapy demonstrated a favorable outcome in studies involving gram-negative bacteria (OR, 0.38; 95% CI, 0.18-0.79). Studies conducted in Europe reported a statistically significant decrease in overall mortality risk with combination therapy (OR, 0.52; 95% CI, 0.32-0.86).

Forest Plot of one-year Mortality of patients with infective endocarditis compared between combination therapy and monotherapy

**Figure:**
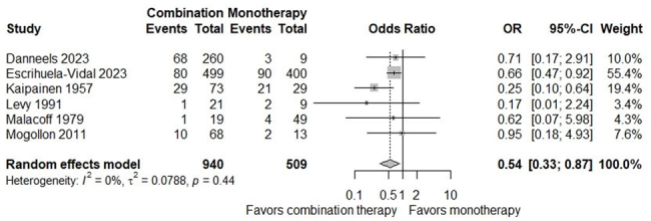

**Conclusion:**

Combination therapy in patients with infective endocarditis (IE) was associated with reduced one-year mortality rates and lower mortality among cases involving gram-negative bacteria. The observed significance in European studies may be attributed to a higher proportion of papers focusing on gram-negative infections and reporting one-year mortality outcomes.

Forest Plot of all-cause Mortality at any time point of patients with infective endocarditis compared between combination therapy and monotherapy (only studies from Europe)

**Figure:**
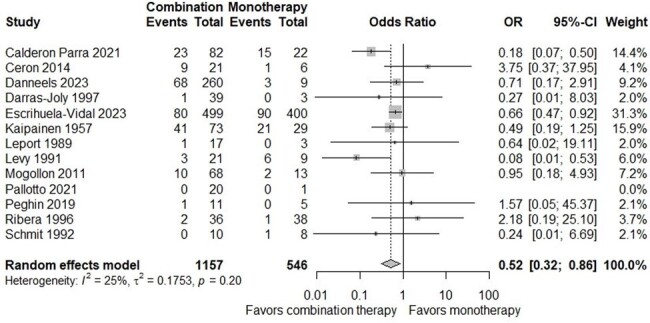

**Disclosures:**

**Vance G. Fowler, MD, MHS**, Affinergy: Advisor/Consultant|ArcBio: Stocks/Bonds (Private Company)|Armata: Advisor/Consultant|Astra Zeneca: Advisor/Consultant|Astra Zeneca: Grant/Research Support|Basilea: Advisor/Consultant|Basilea: Grant/Research Support|ContraFect: Advisor/Consultant|ContraFect: Grant/Research Support|Debiopharm: Advisor/Consultant|Destiny: Advisor/Consultant|EDE: Grant/Research Support|Genentech: Advisor/Consultant|Genentech: Grant/Research Support|GSK: Advisor/Consultant|Janssen: Advisor/Consultant|Karius: Grant/Research Support|MedImmune: Grant/Research Support|Merck: Grant/Research Support|sepsis diagnostics: Patent pending|UptoDate: Royalties|Valanbuio: Stocks/Bonds (Private Company)|Valanbuio: Stocks/Bonds (Private Company) **Joshua T. Thaden, MD, PhD**, National Institutes of Health K08 AI171183 (Thaden): Grant/Research Support

